# The Effect of Upright Posture on Left Atrial Strain in Competitive Athletes

**DOI:** 10.3390/jcdd11090284

**Published:** 2024-09-09

**Authors:** Joscha Kandels, Stephan Stöbe, Robert Percy Marshall, Andreas Hagendorff, Michael Metze

**Affiliations:** 1 Klinik und Poliklinik für Kardiologie, Universitätsklinikum Leipzig, Liebigstr. 20, 04103 Leipzig, Germany; 2 RasenBallsport Leipzig GmbH, Cottaweg 3, 04177 Leipzig, Germany; 3 Department of Orthopedic and Trauma Surgery, Martin-Luther-University Halle-Wittenberg, 06120 Halle, Germany

**Keywords:** transthoracic echocardiography, athletes, upright posture, deformation, left atrial strain, speckle tracking echocardiography

## Abstract

Background: Left atrial strain (LAS) assessment by speckle tracking echocardiography (STE) has been shown to be a remarkable means of quantifying LA function as an early marker of LV pathology. As exercise testing is also performed on a treadmill, the aim of this study was to investigate the effect of upright posture on LAS in healthy athletes. Methods: Fifty male athletes (mean age 25.7 ± 7.3 years) underwent transthoracic echocardiography (TTE) in the upright and left lateral positions. In addition to the conventional echocardiographic parameters, in all athletes, LA conduction strain (LAScd), contraction strain (LASct), reservoir strain (LASr), and maximum LA volume (LAV_max_) were assessed by STE in both positions. Results: Comparing upright posture and the left lateral position, LAScd (−14.0 ± 5.9% vs. −27.4 ± 7.1%; *p* < 0.001), LASct (−4.6 ± 3.5% vs. −11.3 ± 4.1%; *p* < 0.001), LASr (18.7 ± 7.6% vs. 38.7 ± 8.0%; *p* < 0.001), and LAV_max_ (24.4 ± 8.8% vs. 50.0 ± 14.2%) differed significantly. Conclusions: Upright posture has a significant effect on LA deformation, with decreased LAScd, LASct, and LASr. The results of this study contribute to the understanding of athletes’ hearts and must be considered when performing echocardiography in healthy athletes on a treadmill.

## 1. Introduction

Speckle tracking echocardiography (STE) has become an important echocardiographic tool for the assessment of left ventricular (LV) function. Global longitudinal strain (GLS) based on STE has been shown to be an accurate parameter for the early detection of LV dysfunction [1]. Due to the direct interaction between the left atrium (LA) and the LV, assessment of LA function is essential for the accurate assessment of cardiac function. Until the recently introduced LA strain (LAS), LA function was mainly defined by LA size [2].

Based on the cardiac cycle and the three phases of LA function, LAS is composed of three different strain parameters: LA reservoir strain (LASr), which describes passive LA filling by the pulmonary veins during LV systole as LA volume increases; LA conduit strain (LAScd), which describes the phase of early LV filling during diastole as LA volume decreases; and LA contraction strain (LASct), when the LA actively ejects blood into the LV after the passive LV filling [3].

Previous studies have shown that LAS helps to detect functional changes before geometric changes [3,4,5,6]. In a meta-analysis of 2542 healthy subjects by Pathan et al., the normal reference range for reservoir strain was 39% (95% CI, 38–41%); for conduit strain, it was 23% (95% CI, 21–25%); and for contractile strain, 17% (95% CI, 16–19%) [7].

A standardized transthoracic echocardiography (TTE) examination, conducted in accordance with the current recommendations, is performed with the subject in the left lateral position [8]. Nevertheless, for competitive athletes undergoing treadmill testing, TTE can be performed in an upright posture [9]. As previously demonstrated, the upright posture has a considerable effect on LV deformation [10]. Although the impact of upright posture on physiological parameters such as blood pressure and heart rate is well documented [11], the effect of upright posture on LA deformation has not been described in the literature in a cohort of healthy athletes. In a previous study by Gottfridsson et al., controlled maneuvers such as continuous positive airway pressure by mask and a passive leg raise maneuver had no significant impact on LASct measurements in a cohort of healthy subjects [12].

The effect of an upright posture on LA strain has yet to be elucidated. This study aimed to investigate the influence of an upright posture on LA strain in healthy athletes. Based on the observed effects of LV deformation, we hypothesized that upright posture would have a significant effect on LA deformation.

## 2. Materials and Methods

This study included 50 male athletes who underwent TTE as part of their pre-participation screening at the University Hospital of Leipzig between March 2018 and August 2021. All participants were engaged in competitive sports and in training for more than 20 h per week. The athletes were examined by TTE in both the upright and left lateral positions. They provided informed consent after receiving a comprehensive explanation of the purpose and procedures of this study. This study adhered to the ethical standards of the Declaration of Helsinki and was approved by the Ethics Committee of the University of Leipzig (073/18-ek).

TTE was conducted according to a standardized protocol using a Vivid e9 or Vivid e95 ultrasound system with a 4Vc phased array probe (GE Healthcare Vingmed Ultrasound AS, Horten, Norway). To minimize measurement errors, the examiners followed the current guidelines for performing an echocardiographic examination [13]. This includes instrument settings during image acquisition (pre-processing), as well as post-processing for optimal image storage and acquisition. All examinations were performed by a board-certified cardiologist. The data sets were analyzed through post-processing using the EchoPac software (Version 206, GE Healthcare Vingmed Ultrasound AS, Horten, Norway). In all participants, systolic and diastolic blood pressure was measured after a five-minute period of rest in both the upright and left lateral positions.

### 2.1. Conventional Echocardiographic Parameters

The diameter of the left ventricular outflow tract (D_LVOT_) was measured in the left parasternal long-axis view in the left lateral position. Relative wall thickness (RWT) was calculated as twice the left ventricular posterior wall diameter (LVPWD) divided by the left ventricular end-diastolic diameter (LVEDD), as determined using anatomical M-mode in parasternal short-axis view and confirmed by biplane scanning. Left ventricular mass (LVM) was calculated using the Devereux formula and indexed to body surface area (LVMi). The normal range for LVMi was defined as <115 g/m^2^ (males) [14].

The left ventricular outflow tract velocity time integral (VTI_LVOT_) was measured by pulsed wave (PW) Doppler in the apical long-axis view with the sample volume positioned precisely at the D_LVOT_ measurement location. Left ventricular stroke volume (LVSV_Doppler_) was calculated by multiplying the cross-sectional area of the LVOT by VTI_LVOT_. Left ventricular ejection fraction (LVEF), end-diastolic (LVEDV) and end-systolic (LVESV) volumes, and biplane left ventricular stroke volume (LVSV_biplane_) were assessed using biplane Simpson’s rule in the apical 2- and 4-chamber views [14]. In both approaches, left ventricular stroke volume (LVSV) was indexed to body surface area (LVSVi). Left atrial volume index (LAVi) was determined in accordance with current recommendations [14].

### 2.2. Parameters of Left Atrial Deformation

**Figure 1:**
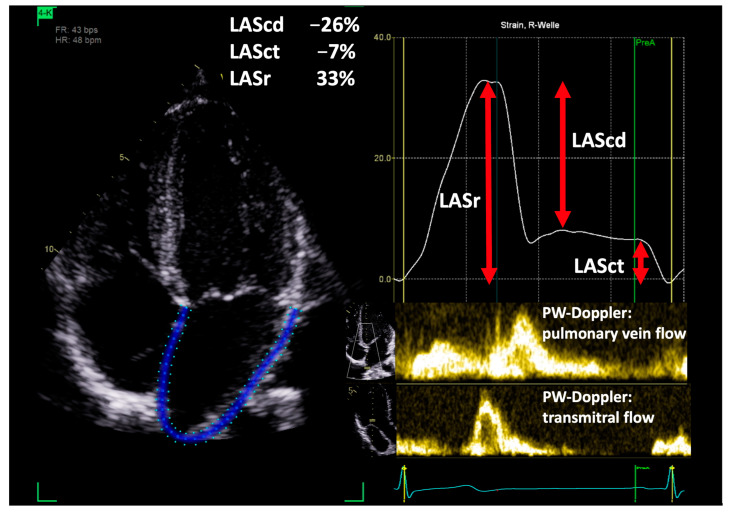
Schematic illustration of the assessment of left atrial strain (LAS): conduction strain (LAScd), contraction strain (LASct), and reservoir strain (LASr) in the apical 4-chamber view in temporal relation to transmitral and pulmonary vein flow.

In all athletes, LAS was assessed in upright posture and left lateral position according to European Association of Cardiovascular Imaging (EACVI) recommendations [15]. LAS was measured in the apical 2- and 4-chamber view as illustrated in Figure 1. The reference time point was the end of LV diastole, determined by transmitral inflow. Measurements were performed by an automated software (EchoPAC software version 206, GE Healthcare). Prior to calculating the data, the examiner was required to verify the correct delineation of the atrial wall and endocardial border. The three phases of the LAS were defined as follows: LASr is the peak strain before mitral valve opening during left ventricular systole; LASct is the strain at the end of ventricular diastole minus the strain at the onset of atrial contraction; LAScd is defined as the difference between LASct and LASr [15]. Based on the apical 2- and 4-chamber view values, the biplane values were calculated by the automated software. Additionally, the maximum LA volume was measured by the software based on the delineation of the atrial wall/endocardial border.

### 2.3. Statistical Analysis

Statistical analyses were conducted using SPSS Statistics version 28.0 (IBM, Armonk, NY, USA). The normality of the distribution was evaluated using the Kolmogorov–Smirnov test. Continuous variables were presented as mean ± standard deviation (SD) and compared between groups using Student’s *t*-test. A significance level of *p* < 0.05 was considered statistically significant. Intra- and interobserver variabilities among 20 patients were evaluated for LAS using the Kappa coefficient (κ) under identical conditions. The second investigator conducted the assessments without knowledge of the results from the initial examination.

## 3. Results

The present study included 50 male athletes, with a mean age of 25.7 ± 7.3 years. The athletes’ baseline characteristics are provided in Table 1.

There was no significant difference in mean systolic and diastolic blood pressure values between the upright and left lateral positions. However, the heart rate (HR) was significantly higher in upright posture (Table 2). Left ventricular volumes and diameters were significant lower in upright posture than in the left lateral position (Table 2). The values for IVSD and LVPWD did not differ significantly between body positions (Table 2). Cardiac output was significantly higher in upright posture compared to the left lateral position, whereas LVEF was similar in both groups (Table 2).

**Figure 2:**
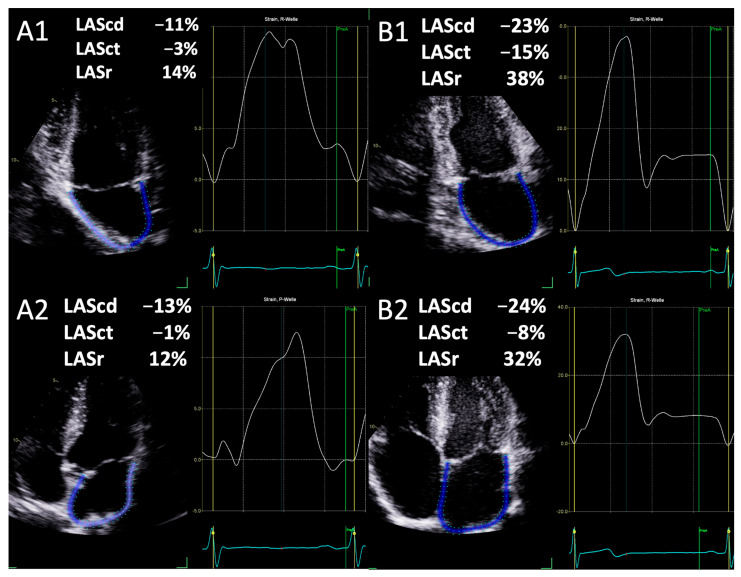
Assessment of left atrial conduction strain (LAScd), contraction strain (LASct), and reservoir strain (LASr) in apical 2-chamber view (**A1**,**B1**) and 4-chamber view (**A2**,**B2**) in upright posture (**A1**,**A2**) and left lateral position (**B1**,**B2**).

The results of the LAS measurements are presented in Table 3. No significant difference was observed between the two- and four-chamber views for LASr, LAScd, and LASct. Yet, a significant difference was noted between them in upright posture and left lateral position as shown in Figure 2 using the example of an athlete’s examination. In addition, LAV_max_ was significantly smaller in upright posture compared to left lateral position (Table 3).

The intraobserver variability demonstrated high agreement for LAS in upright posture (κ = 0.80; z = 4.3, *p* < 0.001) and in the left lateral position (κ = 0.89; z = 4.44, *p* < 0.001). Furthermore, interobserver variability between two investigators also showed good agreement for LAS in both the upright posture (κ = 0.72; z = 4.23, *p* < 0.001) and the left lateral position (κ = 0.85; z = 4.97; *p* < 0.001).

## 4. Discussion

*The main findings of the present study are as follows:*

*(1) Upright posture exerts a significant influence on LA deformation in healthy athletes. (2) LASr, LAScd, and LASct are significantly reduced in upright position in comparison to the left lateral position. (3) LAV_max_ as an indirect predictor of preload is also significantly reduced in upright position.*

Recently, the echocardiographic assessment of LAS has become a highly regarded parameter for the assessment of LA function. In patients with cardiac diseases such as arterial hypertension, a reduction in LAS was observed prior to the enlargement of the LAD or LV [16]. This enabled the early detection of functional changes before the onset of morphologic changes [17]. Telles and colleagues showed in a cohort of 71 subjects with heart failure with preserved ejection fraction (HFpEF) that impaired LAS was associated with increased LA stiffness and abnormal exercise haemodynamics [18]. Torii et al. demonstrated that LAS is a valuable parameter in predicting recovery of LVEF in a cohort in 100 patients with reduced LVEF (<40%) [19]. Saijo et al. also showed that LASct was an independent predictor for exercise intolerance in 532 patients with hypertrophic cardiomyopathy [20]. Cerrito et al. further support the relevance of LA strain as a diagnostic tool for assessing LV diastolic function, particularly in scenarios where traditional metrics such as E/e’ ratio may be ambiguous [21]. Tsai et al. found that decreased LA strain and strain rate were associated with paroxysmal atrial fibrillation, suggesting that LA mechanical function is crucial for maintaining sinus rhythm and preventing AF [22]. Ma et al. demonstrated the predictive value of LA strain in identifying patients at risk of atrial fibrillation recurrence after catheter ablation [23]. In 2021, Sachdeva et al. also emphasized the role of reduced LA strain in predicting stroke in atrial fibrillation patients, further illustrating the broad applicability of LAS as a diagnostic and prognostic tool [24].

This supports our findings that posture-induced changes in LAS should be carefully considered, especially in populations with a predisposition to arrhythmias. Our results align with the existing literature demonstrating the sensitivity of LAS to changes in preload and hemodynamic conditions. Donal et al. emphasized that LA strain parameters are influenced by loading conditions and LV function, and our findings corroborate this by showing significant reductions in LAS parameters when athletes are in an upright position, which likely alters preload conditions [25].

Despite the abundance of research on cardiac disease, there is a paucity of data on LAS in healthy subjects and competitive athletes. The impact of upright posture on LAS in competitive athletes has yet to be examined. In a study conducted by Genovese et al., 25 healthy volunteers underwent TTE during acute stepwise reductions in preload using a tilt table maneuver, during which LAS was measured. The authors reported that LAS was preload-dependent, but to a lesser degree than LAV_max_, and concluded that these findings underscore the diagnostic value of LAS in comparison to LAV_max_ [26]. Our study extends these findings by specifically examining competitive athletes, a population characterized by high cardiac output and adaptive cardiac remodeling. In our study, both LAS and LAV_max_ were significantly affected by upright posture compared to the left lateral position. This difference might be explained by the higher negative effect of upright posture on preload than that of a head-up tilt maneuver. Santoro et al. evaluated the role of LA deformation properties on LV filling at rest and immediately after maximal exercise in competitive water polo players, using speckle tracking echocardiography (STE). Their findings indicated that maximal exercise led to a reduction in atrial longitudinal strain, but all strain rate (SR) parameters increased, highlighting the acute adaptations of the cardiovascular system to intense exercise. This study provides additional context for understanding how dynamic changes in cardiovascular function, such as those induced by posture or exercise, can influence LAS measurements [27]. Park et al. investigated the incidence and determinants of left atrial enlargement (LAE) and its association with LA strains in highly trained university athletes. They found that LAE was relatively common (19.1%) and associated with lower heart rate, higher sports type with cardiovascular demand, lower LV global longitudinal strain (LVGLS), and increased LV stroke volume (LVSV). Despite the association between LAE and lower LA reservoir strain, the incidence of abnormal strain values was low, indicating preserved LA function even in the presence of structural enlargement [28]. This study underscores the importance of differentiating between adaptive and pathological remodeling in athletes.

In the study by Gottfridsson et al., controlled maneuvers including continuous airway positive pressure (CPAP) via face mask and passive leg raise maneuver were performed on 38 healthy subjects. The team asserted that LASct appeared to be preload-independent [12]. These findings are inconsistent with those of our study, which demonstrated that upright posture exerts a significant effect on LAS due to alterations in preload conditions. In a recently published work by Cicetti et al., LAS was examined in 38 ICU patients with circulatory shock before and after fluid administration. The results demonstrated a significant increase in LAS components during fluid administration [29]. In contrast, Park et al. investigated the effect of preload on LAS in 41 subjects who underwent TTE before and after regular hemodialysis. They described that LAS is relatively independent from preload [30]. This difference can be explained with the comorbidities found in patients on hemodialysis, which is mainly hypertension. These patients show reduced LAS [31], and reservoir function might be impaired due to structural changes in the left atrium such as fibrosis [32]. The highly significant differences in LAS in upright posture compared to the left lateral position in our study may be explained by the fact that upright posture and volume changes due to hemodialysis are different loading modalities on LA morphology and function. Further, these inconsistencies in the effects of preload conditions on LAS emphasize the importance of investigating the effects of different positions on LAS.

This study’s findings on left atrial strain during different postures are consistent with previous research showing posture’s impact on cardiac function. Guazzi et al. demonstrated that posture affects venous return and preload, critical for cardiac output during exercise [33]. Bjällmark et al. found significant differences in myocardial (relaxation) velocities between supine and upright exercises, emphasizing distinct hemodynamic responses [34]. Reuss et al. also confirmed that posture significantly influences myocardial function during exercise stress tests [35]. These studies highlight the importance of considering posture when evaluating cardiac function, especially in athletes.

The quality of limit measurements (e.g., LVPWD) depends on image quality and patent pathology. The healthy population in our study had good ultrasound conditions and no LV hypertrophy, which could affect the accuracy of the cardiac dimension measurements [14]. The measured values and standard deviation of cardiac dimensions (e.g., LVPWD) were within the expected range, as previously shown in the study by Thorstensen et al. [36].

The mathematical models used for calculations, such as biplane EF measurement, are typically designed for the supine position and have not been extensively tested in the upright position. The heart’s geometry changes when shifting from supine to upright due to gravity, intrathoracic pressure changes, and variations in venous return. In the supine position, venous return increases, leading to better filling, while in the upright position, the heart aligns more vertically, reducing its overall size slightly due to decreased preload. The heart also shifts from a rounded shape in the supine position to a more ellipsoid shape in upright position [37]. This change results in lower end-diastolic (EDV) and end-systolic volumes (ESV) in the upright position, reflecting altered filling dynamics and reduced venous return. Consequently, stroke volume and cardiac output decrease transiently, but the heart compensates with an increased heart rate, mediated by the autonomic nervous system. However, we acknowledge that no model is perfect, and this introduces some uncertainty. Therefore, we emphasize that the results should be interpreted within the specific measurement conditions.

Gender matters from an anatomical and physiological point of view. Women have smaller hearts than men, but there are also sex differences in microstructural architecture [38]. Female hearts show a larger ejection fraction and a faster rate but generate a smaller cardiac output. In the current study, only male athletes were examined. Left atrial strain seems to have gender-specific reference values. Eck stein et al. report dependencies of LAS by cardiovascular magnetic resonance feature tracking in 183 healthy subjects (97 females). Females had about 20% higher LAS rates, reservoir strain, and conduit strain compared to males. No gender difference was observed for left atrial booster strain [39]. Finally, it seems important to acknowledge potential vendor-specific differences in LA strain measurements due to variations in imaging technology and post-processing algorithms. Pathan et al. highlighted that while significant strides have been made in standardizing LV strain measurements, similar efforts are still required for LA strain [40].

Wang et al. advised against measuring LA strain across vendor platforms, because software LAS measurement results vary widely [41]. This variability highlights the need for clinicians to be aware of the specific equipment and software used when interpreting LA strain measurements. Consistent use of the same manufacturer and software for longitudinal patient follow-up is recommended to minimize variability.

The purpose of LAS measurements in athletes remains to be scientifically elucidated. Echocardiography is a vital tool in the pre-participation screening and ongoing evaluation of athletes, as highlighted by recent guidelines and studies [42,43]. The comprehensive nature of echocardiographic assessment, which includes structural, functional, and morphological evaluations to detect potential cardiac abnormalities that could predispose athletes to adverse events, has been emphasized. Bhatia et al. extends this approach by screening for coronary artery abnormalities [44]. While the measurement of atrial strain is not mentioned in the current guidelines, LA strain analysis may serve several purposes in athletes: (1) early detection of functional changes; (2) assessment of cardiac adaptation (intensive training, e.g., pathological of adaptive LA enlargement); (3) risk stratification; and (4) monitoring over time (e.g., impact of training regimens).

### Limitations

This study only included young and healthy male athletes. This restricts the generalizability of the findings to other populations, such as female athletes, older individuals, or those with cardiac diseases. Additionally, the relatively small sample size of 50 participants may limit the robustness of our conclusions. In addition, only athletes with good image quality were included, which could introduce a selection bias, as those with suboptimal imaging conditions (e.g., due to higher body mass index or other factors) were not represented in this study. Since this was a single-center study, variability in equipment, technique, and environment could affect its reproducibility. This study does not explore other potential confounding factors that might influence preload, such as hydration status, recent physical activity, or nutritional intake, which could impact the results.

## 5. Conclusions

Upright posture has a significant effect on LA deformation in healthy athletes. LASr, LAScd, and LASct are significantly reduced in upright posture compared to the left lateral position. Additionally, LAV_max_ has been proven as a surrogate parameter for the detection of different preload conditions. These results should be considered when performing TTE in competitive athletes in an upright position. Changes in posture, hydration status, and recent physical activity can alter preload, thereby influencing LAS parameters. Understanding this dependency is crucial for the accurate interpretation of echocardiographic results, particularly in settings where preload may vary, such as in athletes during different phases of training or in patients with fluctuating fluid status. Incorporating LA strain into routine echocardiographic screening improves the ability to detect, monitor, and manage potential cardiac problems in athletes, ultimately contributing to their long-term health and performance.

## Figures and Tables

**Table 1:** Baseline demographic characteristics.

Variables (*n* = 50)	
Age (years)	25.7 ± 7.3
Male (%)	100
Weight (kg)	83.9 ± 12.2
Height (cm)	186.2 ± 6.5
BSA (m^2^)	2.08 ± 0.17
BMI (kg/m^2^)	24.1 ± 2.3

BSA = body surface area; BMI = body mass index.

**Table 2:** Hemodynamic and conventional echocardiographic parameters.

Variables	Upright Posture	Left Lateral Position	*p* Value
sBP (mmHg)	128.3 ± 8.3	125.3 ± 9.9	0.104
dBP (mmHg)	74.3 ± 6.8	72.9 ± 12.2	0.482
HR (1/min)	79.1 ± 13.9	61.0 ± 10.1	<0.001
IVSD (mm)	9.9 ± 1.2	9.7 ± 1.4	0.299
LVPWD (mm)	9.6 ± 1.1	9.3 ± 1.2	0.383
LVEDD (mm)	48.2 ± 4.2	54.8 ± 5.0	<0.001
LVESD (mm)	31.9 ± 3.7	34.8 ± 4.7	<0.001
LVEDV biplane (mL)	117.4 ± 29.5	157.9 ± 30.8	<0.001
LVESV biplane (mL)	48.3 ± 15.3	62.7 ± 19.1	<0.001
LVMi (g/m^2^)	98.3 ± 21.0	106.0 ± 16.8	0.072
RWT	0.40 ± 0.05	0.35 ± 0.05	<0.001
LVSV Doppler (mL)	65.3 ± 14.2	94.7 ± 15.8	<0.001
LVSVi Doppler (mL/m^2^)	31.2 ± 6.0	45.4 ± 7.2	<0.001
LVSV biplane (mL)	69.1 ± 16.3	95.1 ± 16.0	<0.001
LVSVi biplane (mL/m^2^)	33.2 ± 7.0	45.7 ± 6.4	<0.001
MV E Max (m/s)	0.78 ± 0.13	0.77 ± 0.15	0.924
MV A Max (m/s)	0.48 ± 0.10	0.40 ± 0.08	<0.001
E/A	1.65 ± 0.26	1.99 ± 0.48	<0.001
LVOT VTI (cm)	15.7 ± 3.1	22.8 ± 3.7	<0.001
EF (%)	59.7 ± 5.3	61.1 ± 5.5	0.197
CO (L/min)	4.72 ± 1.12	5.28 ± 0.98	<0.001
CI ((L/min)/m^2^)	2.26 ± 0.49	2.54 ± 0.43	0.003

sBP = systolic blood pressure; dBP = diastolic blood pressure, HR = heart rate; IVSD = interventricular septum diameter; PWD = posterior wall diameter; LVEDD = left ventricular end-diastolic diameter; LVESD = left ventricular end-systolic diameter; LVMi = left ventricular mass index; RWT = relative wall thickness; LVSV = left ventricular stroke volume; LVSVi = left ventricular stroke volume index; MV = mitral valve; Max = maximum; LVOT VTI = left ventricular outflow tract velocity time integral; EF = ejection fraction; CO = cardiac output; CI = cardiac index.

**Table 3:** Echocardiographic parameters of left atrial morphology and function.

Variables	Upright Posture	Left Lateral Position	*p* Value
4 Chamber View			
Conduction Strain (%)	−13.9 ± 6.5	−26.9 ±6.9	<0.001
Contraction Strain (%)	−4.0 ± 3.3	−9.0 ± 4.1	<0.001
Reservoir Strain (%)	17.9 ± 7.5	34.4 ± 13.4	<0.001
Maximum Volume (mL)	20.8 ± 8.0	41.8 ± 15.7	<0.001
2 Chamber View			
Conduction Strain (%)	−14.0 ± 7.8	−27.7 ± 9.0	<0.001
Contraction Strain (%)	−5.4 ± 4.9	−12.2 ± 9.9	<0.001
Reservoir Strain (%)	19.6 ± 10.4	41.6 ± 9.7	<0.001
Maximum Volume (mL)	27.1 ± 11.0	54.4 ± 17.2	<0.001
Biplane			
Conduction Strain (%)	−14.0 ± 5.9	−27.4 ± 7.1	<0.001
Contraction Strain (%)	−4.6 ± 3.5	−11.3 ± 4.1	<0.001
Reservoir Strain (%)	18.7 ± 7.6	38.7 ± 8.0	<0.001
Maximum Volume (mL)	24.4 ± 8.8	50.0 ± 14.2	<0.001

## Data Availability

The data sets used and analyzed during the current study are available from the corresponding author upon reasonable request.
